# The Importance of Monitoring Neurodevelopmental Outcomes for Preterm Infants: A Comparison of the AIMS, GMA, Pull to Sit Maneuver and ASQ-3

**DOI:** 10.3390/jcm11216295

**Published:** 2022-10-26

**Authors:** Roksana Malak, Brittany Fechner, Marta Stankowska, Katarzyna Wiecheć, Tomasz Szczapa, Joanna Kasperkowicz, Maja Matthews-Kozanecka, Teresa Matthews Brzozowska, Oskar Komisarek, Przemysław Daroszewski, Włodzimierz Samborski, Ewa Mojs

**Affiliations:** 1 Department and Clinic of Rheumatology, Rehabilitation and Internal Medicine, Poznań University of Medical Sciences, 61-545 Poznan, Poland; 2 Department of Clinical Psychology, Poznań University of Medical Sciences, 60-812 Poznan, Poland; 3 Neonatal Biophysical Monitoring and Cardiopulmonary Therapies Research Unit, II Department of Neonatology, Poznan University of Medical Sciences, 60-535 Poznan, Poland; 4 Department of Social Sciences and the Humanities, Poznan University of Medical Sciences, 60-806 Poznan, Poland; 5 Department of Orthodontics and Masticatory Dysfunction, Poznan University of Medical Sciences, 60-812 Poznan, Poland; 6 The Chair and Clinic of Maxillofacial Orthopaedics and Orthodontics, Poznan University of Medical Sciences, 60-812 Poznan, Poland; 7 Department of Plastic, Reconstructive and Aesthetic Surgery, Collegium Medicum in Bydgoszcz, Nicolaus Copernicus University in Torun, 85-821 Bydgoszcz, Poland; 8 Department of Organization and Management in Health Care, Poznan University of Medical Sciences, 61-545 Poznan, Poland

**Keywords:** GMA, pull to sit, NBAS, AIMS, ASQ-3, NSS-8, parent assessment, preterm infant, neurodevelopment, early intervention

## Abstract

Background: Clinicians and parents should closely monitor the neurodevelopment of very preterm infants. The aim of our study was to compare whether neurodevelopmental assessments completed by parents and those done by specialists yielded similar outcomes. We wanted to check whether the assessments completed by specialists and parents were comparable in outcomes to emphasize the important roles of early assessment of a child and of the parents in their child’s treatment and medical care. Another aim was to check whether or not the pull to sit maneuver from the Neonatal Behavioral Assessment Scale (NBAS) is still a parable item in well-known scales of neurodevelopment. Methods: We assessed 18 preterm neonates in the fourth month of corrected age with scales such as the General Movement Assessment (GMA), the Alberta Infant Motor Scale (AIMS), and the pull to sit maneuver from the NBAS. Finally, we asked parents to complete the Ages and Stages Questionnaire, Third Edition (ASQ-3). Results: We found that the respective assessments completed by specialists and parents are comparable in outcomes. We also found that the pull to sit item from the NBAS was still a valid test since it showed similar findings to those from the AIMS, the GMA, and the ASQ-3. Conclusions: The pull to sit item from the NBAS is an important item for assessment of very preterm infants. Specialists should also take into consideration the input and concerns of parents when planning for treatment and intervention.

## 1. Introduction

Children should be screened and monitored in respect to their neurodevelopment. The American Academy of Pediatrics’ (AAP) developmental screening policy states that the “Early identification of developmental disorders is critical to the well-being of children and their families” [1]. The early identification of any developmental problem is especially important for very preterm infants since it enables a clinician to apply early intervention strategies that are important for preterm neonates and their parents. This explains why motor repertoire should be assessed by methods such as Prechtl’s General Movements, (GMA) which has a very high sensitivity of about 82 to 100% for infants at three months post-term [2]. Postural patterns typical for this age may also be included in the assessment. Early detection of problems related to motor repertoire enables the clinician to apply proper early intervention [3]. Overall, very preterm infants present with poorer scores in their movement repertoires, observed postural patterns, and movement character [3]. Parents of neonates hospitalized in the neonatal intensive care unit (NICU) usually experience a sense of powerlessness due to preterm birth [4]. They may feel helpless due to a lack of adequate knowledge about how to interact with their infants in the NICU. This may then lead to adverse parent outcomes after hospitalization, such as depression and other dysfunctional parenting patterns [5,6,7,8]. Monitoring their infant’s health and behavior may empower parents in the everyday care of their child [9,10]. The best way to empower a parent is by having the parent assist with the early assessment of their child’s neurodevelopment. Therefore, there are special scales which enable them to do so, such as the International Classification of Functionality (ICF) and The Ages and Stages Questionnaire, Third Edition (ASQ-3).

This is extremely important, since neonates who are born at less than 32 weeks of gestational age and with a birth weight of less than 1500 g are at greater risk of having development delays [11]. The identification of the aspects that are delayed may help in applying appropriate therapeutic interventions that focus on improving special skills for these infants. Infants who are born preterm are more likely to have severe impairments, such as physical impairments, but a also to perform poorly regarding school-age function as a long-term risk factor [12]. Regarding short-term outcomes, more than a million preterm infants have died in the first month of life due to severe complications associated with premature birth [13,14]. Other short-term outcomes include intraventricular haemorrhage (IVH), necrotising enterocolitis (NEC), bronchopulmonary dysplasia (BPD), and retinopathy [15]. This is difficult because it tends to make parents of preterm children feel helpless, afraid, worried, powerless, guilty, and stressed. Therefore, the neurodevelopment of neonates should be assessed, monitored, and facilitated—firstly, in order to improve the development of the preterm child, and secondly, to help professionals empower parents [16]. There are many methods that can be used to detect difficulties or delays in a preterm neonate’s psychomotor development. It is important for a clinician to carefully consider the best methods that are both valid and useful for assessing very preterm neonates. We would like to describe the standardized assessments in the following order: The General Movement Assessment (GMA), the Alberta Infant Motor Scale (AIMS), the pull to sit maneuver from the Neonatal Behavioral Assessment Scale (NBAS), and the Ages and Stages Questionnaire, Third Edition (ASQ-3).

One such assessment is Heinz F. R. Prechtl’s GMA, which focuses on the movement repertoire of infants, and which has a high predictive power for the neurodevelopment of preterm and full-term infants presenting with various risk factors [17,18]. A reduced motor optimality score from the GMA is often associated with more severe motor impairments, even a few years later [17,18]. The GMA has a specificity of 91% (95% CI 83–95) for general movements (GMs) in prognosticating cerebral palsy. This is comparable to a cranial ultrasound, magnetic resonance imaging (MRI), and a neurological examination. GMs may be observed from early fetal life up to three months and no further than five months corrected age [17,18]. According to GM type, writhing movements are the first movements that appear in fetal life until two months corrected age after birth in typically developing infants [19]. Another type of normal GM is fidgety general movements. These are small and tiny movements most prominent between 11- and 16-weeks corrected age [12]. Types of GMs observed in atypically developing children, so-called abnormal GMs, include cramped synchronized (CS GMs), poor-repertoire (PR GMs), and chaotic (Ch GMs). Abnormal GMs, especially CS GMs, are some of the most reliable indicators of neurodevelopmental disorders [18].

Among the standardized assessment methods used to detect motor developmental abnormalities in at-risk preterm infants there is also the AIMS [12]. The AIMS enables clinicians to assess not only the level of motor development but also the quality of movement in infants [12]. The AIMS can be used in the assessment of children from birth until 18 months of life corrected age [19]. The AIMS consists of 58 items. In this assessment, the examiner observes an infant in the prone (21 items), supine (9 items), sitting (12 items), and standing (16 items) positions. The AIMS is one of the best predictive scales of atypical infant development [20]. The best sensitivity and specificity of the AIMS was recently observed at 4 months corrected age when a score below the 10th percentile provided a sensitivity of 77.3% and a specificity of 81.7% [20]. 

Another scale used to assess the neurodevelopment of preterm infants is the NBAS. It considers factors such as habituation, social interaction, the motor system, state regulation and organization, the autonomic nervous system (ANS), reflexes, and supplementary items [21]. The NBAS differentiates between six different types of behavioral states: deep sleep (I), light or active sleep (II), awake—drowsy (III), alert (IV), alert and active with increased motor activity (V), and crying (VI). The maximum sensitivity and specificity of the NBAS assessment of states were between 71.7 and 51.2%, respectively [22]. The NBAS can be used for infants up to 8 weeks postmenstrual age. According to Canals et al. (2011), the higher the NBAS scores in most of the items, the less the maternal anxiety that was observed in the 12th month [23]. Similarly, if mothers were less anxious during pregnancy, then most of the NBAS scores, for instance, Orientation Processes (Social—Interactive) and State—Organization, were assessed in neonates as ‘optimal’ [24]. We used the NBAS in previous studies and showed parents important aspects to consider in the everyday care of their child, including feeding [21,25,26,27]. In the present study, we preferred to only use the pull to sit item from the NBAS since Thomas Berry Brazelton, the creator of the NBAS, had described this assessment in detail. The NBAS, specifically the pull to sit item, may be a useful predictor of later developmental disabilities [28]. Furthermore, it measures axial tone of the neck and trunk and any appendicular tone of the shoulder, arms, and lower extremities [29]. 

Finally, another scale used to assess the neurodevelopment of preterm infants is the ASQ-3, which assesses five domains of development, including: gross motor, fine motor, problem-solving, personal-social, and communication. According to Fauls et al. (2020), “Domain scores are compared to normative scores, and cut-off points rate development in that domain as either ‘typical’ (<1 SD below mean), ‘monitor’ (≥1 SD to <2 SD below mean), or ‘refer for further assessment’ (‘refer’) (≥2 SD below mean)” [30]. Parents were asked to complete this developmental screening tool so they could trust their own observations and later present the results from this questionnaire to a specialist. The ASQ-3 may be used for children aged 0 to 66 months. This assessment is also valid in the identification of gross motor difficulties. The AAP reports that the ASQ-3 has a sensitivity range of 0.70 to 0.90 and a specificity range of 0.76 to 0.91 (the values above the 0.70 threshold represent a high sensitivity and specificity) [31]. The test-retest reliability of the ASQ-3 is 92%, with a sensitivity of 87.4% and a specificity of 95.7% [1].

The aim of our present study was to check whether the results from the ASQ-3 assessment completed by parents and the results from the same assessment completed by specialists produced similar outcomes. We decided to compare the assessments by parents to those by specialists in order to emphasize the important role of parents of preterm infants, who usually experience a sense of lack of control or powerlessness. Including parents as an important component in the care and treatment of their preterm child may help them in establishing full responsibility their care. Neurodevelopmental scales used by specialists (the GMA, the pull to sit maneuver from the NBAS, and the AIMS) as well as those used by parents (ASQ-3) help to detect children who are at risk of developmental delay. We did not want to show any particular delay in children but rather, to show that parents and specialists alike can detect an overall general delay in development. Another aim was to check to see if the pull to sit maneuver from the NBAS, which is the test performed by many pediatricians, neonatologists, and neurologists, is comparable to the assessment by parents.

## 2. Materials and Methods

The key objective of our study was to compare the assessment results of parents and specialists as well as to empower parents caring for their preterm infants. Due to our study taking place during the COVID-19 pandemic (May/June 2021), we included 18 infants in our study, but we managed to assess only 16 out of 18 of these preterm infants who were hospitalized at the Gynecology and Obstetrics Clinical Hospital. Inclusion criteria included infants born before the 32nd week of gestation and with a birth weight lower than 1500 g. All neonates presented with feeding problems. All parents provided us with their written consent to perform the examination of their child. The exclusion criteria included oxygen desaturation (SpO2 < 88%), a heart rate lower than 100 or higher than 205 beats per minute, active inflammation, sepsis, bone replacement, tumors, encephalopathy, hypotension, and lethal birth defects (e.g., Edwards, Patau, etc.) 

We described the assessment of this same sample in a previous article [25]. We performed the second examination of these infants when they were in the fourth month of corrected age. The average age of gestation was 29 ± 2 weeks (minimum 25 weeks postmenstrual age and maximum 32 weeks postmenstrual age). The average birth weight was 1230 g ± 229 g. The average gestational age of the infants during the time of assessment was 4 months corrected age (6months calendar age). Most of the children in our study received an Apgar score of 8 points (min. 5, max. 9). The average value of the umbilical cord arterial pH was 7.23 (SD ± 0.13). Demographic data has been added below (Table 1).

We decided to observe infants in the fourth month of corrected age since this time period has a prognostic value [1,11,32,33,34,35,36]. Assessing GMs in the third to fourth month of corrected age may enable an examiner to determine increased risk factors for the development of minor neurological deficits, attention deficit hyper-activity disorder (ADHD), and maladaptive behavior even in 4- to 9-year-old children [35].

### 2.1. Methods

There were three tests (the GMA, AIMS, and ASQ-3) as well as the pull to sit maneuver from the NBAS which were performed over the span of two days by one qualified specialist who graduated from a course of each scale. The first test was the GMA, the second was the AIMS, and the third was the pull to sit maneuver from the NBAS. The next day parents completed the ASQ-3 in the presence of the specialist who had the knowledge to explain to parents how to complete the questionnaire.

We did not check the inter-rater reliability of these tests since one specialist completed the assessments for the study group and the intra-rater reliability of these methods had been described in previous studies. For instance, the intra-rater reliability for the GMA was 85.4% ± 0.1% and 95.4% ± 0.1%, respectively, for the two assessors [37] [for all AIMS scores, the observed intra-rater reliability was more than 75% [38] Lackovic]; for the Brazelton scale—NBAS it was 81% [10.1590/1984-0462/2021/39/2020034] and for the ASQ-3 it was approximately 98% for total ASQ-3, and the intra class correlations between the gold standard and examiners of the ASQ-3 Gross motor was approximately 97–99% [39].

Infants were first assessed using the GMA, a noninvasive method designed by Prechtl. A clinician who had graduated from an elementary and advanced course in the GMA performed the observation and assessment of each infant which lasted approximately 4 min. When considering the GMA, we observed whether a child presented with normal GMs, such as fidgety movements, or abnormal GMs, such as PR GMs, CS GMs, or Ch GMs [40].

The AIMS was the next test performed and was based on the observation of movements. Participants scored 1 point for each observed item and 0 points for a lack of movement. In order to perform the AIMS, a specialized course is not formally required. However, the clinician who performed this assessment for the purposes of this study is experienced in conducting the AIMS, and this was explained and elaborated upon in our previous study [41].

A qualified clinician, having taken the formal NBAS training course provided by the Cambridge Brazelton Centre in the United Kingdom, administered all the aforementioned scales as well as the pull to sit item of the NBAS Motor System cluster. Because of the age of the children (in the fourth month of corrected age), the clinician only performed the pull to sit item from the NBAS, and this maneuver lasted approximately one minute per participant. The NBAS is intended for children who are not older than eight weeks, but the pull to sit is normally performed during most pediatric and neurological visits. The notable head lag, especially during the first few weeks of life, is typical and normal until 3–4 months [42]. Therefore, we think that this concrete probe is more important in children who are older than 8 weeks because head lag should be absent by 3 to 4 months of age when infants typically experience an increased ability to control their neck muscles [42].

To perform the pull to sit maneuver, the clinician grasps the infant’s wrists in order to slowly pull him up from a supine into a sitting position [33]. We expected that in typically developing infants, the head would follow in line with the body when actively lifted, and that the arms would be moderately flexed at the elbows [33]. The score we gave was based on the description of the pull to sit item from the Motor System cluster of the NBAS since it contained many important details. The examiner considers the following factors for this maneuver: how a child attempts to bring his head forward; if a child brings his head to the midline; if it is possible for the child to maintain his head at the midline; how many seconds he could hold his head up; if the shoulder tone increases and neck tone is observed. The scoring system for this item is based on a 0-to-9-point scale, in which a higher number of points indicates better performance from the infant, including smoother movements, such as better head control, and limbs and trunk reaction in the pull to sit item. A lower score is due to lack of head control with no attempt to bring it up, flaccid muscle tone, and no observed shoulder and neck tone. 

Finally, parents were asked to complete the ASQ-3 when their children were in the fourth month of corrected age. They answered each question of the questionnaire on a different day from the formal assessment performed by the specialist. However, they still completed the ASQ-3 in the presence of a specialist. The ASQ-3 was performed at home where parents could calmly complete all the tasks with the children (without the pressure of an unfamiliar environment such as at a clinic or office) and answer the items asked in the questionnaire. This scale is divided into the above-mentioned 5 domains (i.e., fine motor, gross motor, communication, problem solving, personal social). Each domain consists of 6 questions, to which the parent responded “yes” if the child performed a given activity, “sometimes” if he performed it rarely, or “not yet” if the behavior was not present. For all answers, “yes” was given 10 points, “sometimes” was given 5 points, and for the answer “not yet” no points were scored. A child could receive up to 60 points maximum in each cluster. Each parent completed the questionnaire in an average of 12–18 min. The results were then transferred to a table with ranges assessing the child’s development based on the cumulative points from all five domains. Although the ASQ-3 consists of 5 domains, only the gross motor and fine motor domains were compared with the specialist’s assessment. Those two clusters of the ASQ-3 refer to motor development as well as to the GMA, pull to sit maneuver from the NBAS, and AIMS.

Due to the differences in the type of measurement scales we used (nominal/ordinal/quantitative), all methods had to be reduced to one common measurement scale, which was the nominal scale. Each method mentioned contains qualitative items that have been quantified. For instance, a patient can receive zero points in the pull to sit maneuver from the NBAS if he cannot hold his head up independently, and one point if he can hold his head up without a problem, while his legs and arms remain active during the assessment. Each method was classified such that a value of 1 was correct, while a value of 0 was incorrect. The AIMS, the original results of which were presented in percentiles, was converted based on guidelines, where results less than or equal to the 25th percentile indicated abnormal development, and the 26th percentile and above indicated normal development [43]. In our present study, only one element from the NBAS was used—the pull to sit maneuver. The pull to sit maneuver of the NBAS and the ASQ-3 belong to a group of comprehensive tests, consisting of many separate components forming a whole. In order to use the results obtained from these two scales, the responses to them should be properly classified. Based on the recorded results in the zero-one system, clusters based on the similarity of responses were designated with the determination of Euclidean distances. The assignment of patients to individual clusters was based on combining common casings into groups. 

In reference to assessing parental empowerment, we asked the following question from the Neonatal Satisfaction Survey (NSS-8): To what extent do you feel confident with managing the necessary follow-up care of your child/children after coming home? (i.e., breast feeding/nutrition, administering medication). Each parent could answer as either 1 point—“not at all”, 2—“to a small extent”, 3—“to some extent”, 4—“largely”, and 5—“to a very large extent”. When a child was hospitalized in the NICU, a parent completed all 51 questions of the NSS-8. The NSS-8 enables practitioners to measure the experiences of parents in eight spheres of care of their child: (1) “Your overall impression”, (2) Doctors, (3) Visit, (4) Facilities, (5) Siblings, (6) Information, (7) Parental anxiety, and (8) Discharge. Since the described assessment referred to the period of time when a child was discharged, we considered only the eighth sphere of care in our study.

### 2.2. Analytic Strategy

Statistical analysis was performed using Statistica Version 13 software (TIBCO Software, Tulsa, OK, USA). The difference between scales was measured using Cochran’s Q test and Dunn’s Test. Cochrane’s test was used for nominal dependent variables with more than three measurements. For the post hoc test we used Dunn’s test. A *p*-value of <0.05 was considered statistically significant. This study was approved by the Bioethics Committee, consent ref. No. 481/21.

## 3. Results

In reference to the GMA, eight of the children presented PR GMs (8 infants), six children presented normal fidgety GMs, and two infants presented with CS GMs.

The average value obtained from the AIMS was the 72nd percentile (SD ± 19). There were no children who presented with a score lower than the 10th percentile, which meant that there were no scores indicating a risk for neuromotor impairment in the fourth month according to the AIMS [44]. 

According to the pull to sit item probe, we used the descriptive method of scoring based on the NBAS in order to detect all abnormalities. The median score of this item was 7 (the lower Q = 6, the upper Q = 8; min. 2, max. 9). 

Parents also described their children using the ASQ-3. On average, results from the gross motor domain included a score of 51 (SD ± 15) and for the fine motor domain a score of 48 (SD ± 12). For the gross motor domain two infants needed further assessment from the clinician (“refer range” > 2 SD); three infants should be involved in learning activities with parents where parents are taught how to position their child during everyday care, and encourage “tummy time” (putting a child down and lying him on his stomach with his weight on his forearms) under observation from a clinician (“monitor range” 1–2 SD below the mean); and the gross motor development of 11 infants appeared to be normal (“typical range” < 1 SD). For the fine motor system domain, two infants needed further assessment from the clinician (“refer range” > 2 SD), two infants were prescribed learning activities with parents and should be monitored (“monitor range” 1–2 SD below the mean); and for the fine motor development of 12 infants, results appeared to be normal (“typical range” < 1 SD). We then analyzed the motor functioning of the children (Table 2) to determine if there were any statistically significant differences between results from the AIMS, GMA, and pull to sit probe from the NBAS (Table 3), through the use of Dunn’s Test.

We also compared the AIMS, the pull to sit item from the NBAS, and the ASQ-3 for any differences. Similarly, Dunn’s test also revealed that there was no statistically significant difference between the results derived from the AIMS, GMA, pull to sit item from the NBAS, and ASQ-3 (*p* > 0.9999999).

According to Cochran’s Q Test where *p* > 0.05, we found that there was no significant difference between each of the compared scales (Table 4).

## 4. Discussion

The motor development of very preterm infants should be closely monitored. The observation of preterm children should include personal factors such as lability of states, peak of excitement or even temperament, body function, and structured domains based on atypical child development. These assessments, which are based on the biopsychological model, such as the International Classification of Function (ICF) and the NBAS, are recommended as a good tool to estimate a child’s level of functioning, disability, and health [45]. Although preterm infants are at a greater risk for developmental delay, and should receive regular assessment, they do not always benefit from systematic follow-up [46]. The AAP recommends performing ongoing developmental surveillance supplemented with standardized screening tools at specified ages in order to increase the accuracy in detecting developmental disorders. 

It is generally well-known that the GMA and AIMS should be used in everyday practice. We wanted to see whether standardized scales of motor development provided similar results across both clinicians and parents. We wanted to compare the results of motor scales (the GMA and AIMS), and the pull to sit item from the NBAS with the ASQ-3. We decided to assess infants by all aforementioned methods in the fourth month of corrected age as other researchers have done [1,35,47]. Around the fourth month of corrected age, detecting atypical motor function is associated with neurological impairment, and is also well-recognized as a prognostic factor for ongoing disability [34]. Atypical motor function is an item found in many standardized scales such as the AIMS and NBAS. This then helps us to quantify relative head movements of an infant in relation to his or her trunk [48]. When the examination is performed in a typically developing infant, an infant should lift his or her head along with the torso. If a child presents with abnormal development, it would be impossible for him to lift his or her head along with the torso [48]. Abnormal performances on the pull to sit item of the NBAS have predictive significance throughout infancy in diagnosing developmental problems such as atypical outcomes of development (93%–100%), spasticity, cerebral palsy (a positive predictive value for CP is around 83%–88%), or autism spectrum disorder (ASD) (55%) [33].

Similarly, we showed that a parent’s observation of his or her child’s development is also valid and comparable to the assessment made by specialists. This information, as well as that from the ASQ-3 should help parents feel more empowered when interacting with their children. Parents of preterm infants experience a higher incidence of anxiety disorders along with altered parent-infant interactions which negatively impact their children [5]. Currently, popular and sometimes non-evidence-based information found online may make parents less aware and observant of certain important neurobehavioral signs from their infant and may exaggerate their anxieties and uncertainties. False-positive information may cause distress for some parents [49]. Online descriptions of any disorder may increase parental anxiety [50]. Rather, questionable information from the nternet could make some parents of preterm infants feel more anxious and depressed [51]. This is why the value of showing parents that it is worth it to observe their child and that their observations are comparable to that of the assessment of specialists should be more broadly acknowledged. Thanks to the ASQ-3, observing their child helps parents feel self-efficacious and certain that what they observe should be broached with a specialist, and vice versa. 

For instance, the ASQ-3 has been one of the scales with the highest sensitivities and specificities in detecting developmental delay based on research from 2002 to 2009 [52,53,54]. In many publications it has been reported that the ASQ-3 is highly accurate in detecting underlying problems in children with certain risk factors [49,50,51]. Therefore, parents who detect problems in their child by their observation may systematize them by completing the ASQ-3. Seeing that a child presents any health problem, a parent may look for help from and make an appointment with a specialist in order to attain early intervention for their child [46]. The early detection of developmental problems in infants enables timely treatment with effectiveness [55].

The present study shows that specialists in neonatal care should seriously take into consideration what parents observe and say about their preterm infant during a visit to a clinician. The ASQ-3 helps make parents feel empowered. Empowering interventions and programs have a positive impact on the mental health of parents, especially mothers. These programs, containing infant behavior information, have been shown to reduce negative parent experiences such as stress, anxiety, or depressive symptoms, and have also produced self-efficacious and proper caregiving behaviors in parents. Establishing positive partnerships between clinicians and parents may help parents become better aware of their children’s development, alert them to any problems and concerns their child may present, help in the creation of goals and therapy plans, as well as provide an opportunity for parents to receive feedback from professionals about their efforts [51].

This is the second study where we found that if parents are confident and empowered in caring for their preterm child, there are many benefits. In our previous research we showed that 100% of children from the study group did not have to be fed via a tube following the provision of parental education on how to position a child, which pacifier on a bottle to use, and how to successfully facilitate breastfeeding [25]. Similarly, these same parents after six months of care, when their children were in the fourth month of corrected age, still felt confident in caring for their children in everyday life, referring to the answers from the NSS-8 questionnaire.

In a future study, we would like to see how very preterm infants not only develop their motor skills but also their psychosocial abilities. Using the ASQ-3 we would like to compare the problem-solving skills from this questionnaire as well as communication and personal-social skills. According to Hardy et al. (2015) and Vanvuchelen et al. (2017), the ASQ-3 is helpful in screening children at risk for developing ASD [55], [56]. This is important since very or extremely preterm infants are at a greater risk of developing ASD [57,58,59]. We would like to show that simple methods such as these may help to identify at-risk children and therefore allow them to access interventions earlier in life. 

One limitation from our study was that the sample was small and not randomized due to the study occurring during the COVID-19 pandemic (May/June 2021). Further-more, we would like to investigate whether parents would be open to checking their children’s development using standardized developmental scales, such as the ones described here in our study.

## 5. Conclusions

In our study, we found that assessing motor development, including the pull to sit item of the NBAS, is important for very preterm infants. Furthermore, we showed that specialists should consider parental feedback and input. First, parent assessment is comparable to that of specialists, and second, specialists help make parents feel empowered, which can in turn build a parent’s self-confidence with their infant’s caregiving in everyday life. Parents, as the direct facilitators of their children’s upbringing, should be active and continuous participants in their assessment and care.

## Figures and Tables

**Table 1:** Demographic data of the participants.

Average age of gestation		29 ± 2
Average birth weight		1230 g ± 229
Delivery	Natural	6
	Cesarian section	12
Apgar Score		8
Umbilical cord arterial pH		7.27 ± 1.82
Domicile	Town	13
	Village	5
Education of mothers	Master’s degree	9
	Vocational school education	4
	Secondary general education	5
Education of fathers	Master’s degree	6
	Vocational school education	8
	Secondary general education	4

**Table 2:** Motor functioning of the participants.

GMA	Cramped—synchronized	Poor—repertoire	Fidgety
	2	8	6
ASQ-3	Refer ranges	Monitor—ranges	Typical ranges
	2	3	11
Pull to sit (NBAS)	<4 points	3–6 points	7–9 points
	3	3	10
AIMS	72 ± 19.2 percentile		

**Table 3:** Difference between the AIMS, GMA and pull to sit item, Dunn’s Test *p* = >0.999999.

*p*-Value	AIMS	GMA	Pull to Sit Maneuver	Gross Motor (ASQ-3)	Fine Motor (ASQ-3)
AIMS		1	1	1	1
GMA	1		1	0.42	1
Pull to sit maneuver	1	1		1	1
Gross motor (ASQ-3)	1	0.42	1		1
Fine Motor (ASQ-3)	1	1	1	1	

**Table 4:** Cochran’s Q Test for the AIMS, GMA and pull to sit probe.

ANOVA Cochran’s Q Test	AIMS, GMA, Pull to Sit, ASQ-3
Significance level	0.05
Number of infants	16
Degrees of freedom	3
Statistics Q	5.48
*p* value	0.14

## Data Availability

Data are available only under the permission of authors and parents of infants described in the manuscript.
